# Multistage Adsorption–Filtration of Cow’s Milk for Heavy Metal and Radionuclide Removal with Preservation of Milk Quality

**DOI:** 10.3390/foods15193413

**Published:** 2026-09-24

**Authors:** Aray Bazanova, Aitbek Kakimov, Marzhan Tashybayeva, Alibek Muratbayev, Nazerke Muratzhankyzy, Nadir Ibragimov

**Affiliations:** Research School of Food Engineering, Shakarim University, 20A Glinki Street, Semey 071412, Kazakhstan; bibi.53@mail.ru (A.K.); marzhan06081990@gmail.com (M.T.); mr.muratbayev.a@gmail.com (A.M.); nazerkemuratzhankyzy1990@gmail.com (N.M.); ibragimnk@mail.ru (N.I.)

**Keywords:** cow’s milk, adsorption–filtration, heavy metals, radionuclides, taurite, natural zeolite, activated carbon

## Abstract

This study evaluated a modular sorbent-based filtration system for reducing heavy metals and anthropogenic radionuclides in cow milk and assessed changes in selected physicochemical parameters. Taurite (shungite), natural zeolite, and coconut-shell activated carbon 207C were tested in single- and multicolumn configurations at 100 and 150 cm^3^ per sorbent layer. Pb and Cd reduction were evaluated in fortified milk, while pre-existing ^137^Cs and ^90^Sr were assessed in milk from two locations. Milk-quality parameters were measured in separate milk batches. At 150 cm^3^ per layer, taurite→activated carbon reduced Pb and Cd by approximately 90.6% and 91.9%, respectively, to 0.032 and 0.025 mg/kg. Under the 150 cm^3^ radionuclide-testing conditions, zeolite→activated carbon reduced ^137^Cs and ^90^Sr by 75.73% and 73.97%, yielding residual activities of 0.2534 and 0.1123 Bq/kg. The complete three-stage configurations achieved approximately 79.9% and 77.7% reductions, respectively. In the separate quality assessment, taurite→activated carbon produced limited changes in basic composition and viscosity, whereas zeolite-containing configurations caused greater decreases in Na and K. Radionuclide results were interpreted within each source-specific series because milk source and sorbent loading varied simultaneously. Overall, the findings demonstrate substantial laboratory-scale contaminant reduction and support selecting sorbent configurations according to contaminant type, with taurite→activated carbon providing a favourable balance across the separately assessed removal and milk-quality criteria.

## 1. Introduction

Cow’s milk is an important dietary component providing high-quality proteins, lipids, carbohydrates, minerals, vitamins, and other nutritionally important constituents [1,2,3]. Because milk and dairy products are consumed, their chemical and radiological safety is of considerable public-health importance. Milk can be contaminated through polluted soil, feed and drinking water, industrial emissions, agricultural activities, and contamination during production, processing, or packaging [4,5,6]. Transfer of toxic elements through the soil–feed–animal–milk pathway is therefore an important route of contamination requiring systematic monitoring [5,6].

Among inorganic contaminants, lead (Pb) and cadmium (Cd) are of particular concern because of their toxicity, environmental persistence, and potential for food-chain transfer [5,6]. They can accumulate in agricultural soils and vegetation and enter livestock through contaminated feed and water, resulting in their occurrence in milk [6,7]. Major anthropogenic sources include mining and metallurgical activities, industrial emissions, waste disposal, traffic-related pollution, and agricultural inputs [6,7]. Both Pb and Cd are associated with adverse effects following chronic exposure, while Cd is especially concerning because of its bioaccumulative behavior and established carcinogenicity [5,7,8]. Consequently, controlling Pb and Cd in raw milk is important for minimizing dietary exposure and associated health risks [8,9].

Anthropogenic radionuclides represent another persistent group of contaminants relevant to dairy safety. Cesium-137 (^137^Cs) and strontium-90 (^90^Sr) are of particular importance because they can migrate through soil–plant–animal pathways and enter the human diet through contaminated foods. Kazakhstan has a distinct radiological history associated with nuclear testing at the former Semipalatinsk Test Site, and residual contamination in affected territories remains an environmental and food-safety concern [10]. ^137^Cs can be transferred from contaminated soil to plants, animal feed, and milk, whereas ^90^Sr is particularly relevant because of its chemical similarity to calcium [10,11].

Removal of Pb, Cd, ^137^Cs, and ^90^Sr from milk is technically challenging because milk is a structurally complex multiphase matrix. It contains casein micelles, whey proteins, fat globules, lactose, colloidal calcium phosphate, soluble minerals, and other interacting constituents [12]. Pb and Cd have been shown to interact with functional groups of casein and other dairy-matrix components, and their behavior differs between water, casein, and milk systems [13]. The possible interference of native ions such as Na^+^, Ca^2+^, and Mg^2+^ remains unresolved [13], while proteins and colloidal components can contribute to fouling and filtration resistance [14]. These matrix effects limit direct extrapolation from simple aqueous systems and support evaluation of decontamination directly in milk.

Several physicochemical methods have been investigated for contaminant removal from liquid matrices, including membrane filtration, reverse osmosis, chemical precipitation, ion exchange, oxidation, and adsorption [15]. Adsorption is attractive because of its relatively simple operation, broad applicability, and potential use of inexpensive natural materials [16,17]. Its efficiency depends on sorbent affinity and capacity, contact time, operating conditions, material cost, and matrix composition [18,19]. Natural zeolites are attractive because of their abundance, porous aluminosilicate structure, and ion-exchange properties [17,20], whereas activated carbon provides a highly developed porous structure and surface-active sites for heavy-metal adsorption [19]. Their differing physicochemical properties suggest potential complementary effects when applied sequentially, although this remains insufficiently evaluated in complex food matrices.

Adsorption efficiency is affected by sorbent properties, loading, and operating conditions [17,18,19,20]. In a multistage system, these factors must also be considered with bed configuration and hydrodynamic performance. Sequential arrangements may combine ion-exchange, surface-adsorption, and polishing functions, but greater sorbent loading or more beds may increase filtration time, hydraulic resistance, and sorbent consumption. Optimization therefore requires simultaneous consideration of contaminant removal, filtration performance, and milk quality.

Previously, an experimental bench was developed and patented for filtration of liquid food products using different sorbents [21]. The system was subsequently modified to allow several sorption columns to be connected in series or parallel, enabling variation of sorbent combinations, bed configurations, and operating conditions [22]. This configuration provides a platform for investigating multistage adsorption–filtration of cow’s milk.

Despite extensive research on heavy-metal removal, important knowledge gaps remain. Most adsorption studies have used water or simplified model solutions, although direct milk decontamination has been demonstrated using *Saccharomyces cerevisiae* for Cd biosorption [23] and lactic acid bacteria for Pb removal [24]. These studies show that contaminant removal from milk is feasible but primarily concerns biological sorbents rather than sequential mineral–carbonaceous filtration. Information remains limited on how sorbent combination, loading, and bed arrangement influence removal efficiency, hydrodynamic performance, and milk-quality preservation. Moreover, Pb and Cd and the radionuclides ^137^Cs and ^90^Sr are generally investigated separately, and their treatment within a common multistage filtration platform remains insufficiently characterized.

The novelty of this study lies in the integrated evaluation of a multistage adsorption–filtration system based on natural mineral and carbonaceous sorbents directly in cow’s milk. Different sorbent configurations and loadings were assessed while considering contaminant reduction, hydrodynamic performance, and preservation of key milk-quality characteristics. Pb and Cd in experimentally contaminated milk and ^137^Cs and ^90^Sr in naturally contaminated milk were evaluated using a common filtration platform.

Accordingly, this study aimed to evaluate a multistage sorbent-based filtration system for reducing Pb and Cd in experimentally fortified cow milk and pre-existing ^137^Cs and ^90^Sr in non-spiked milk, alongside a separate assessment of changes in selected milk-quality parameters. The objectives were to compare filtration configurations in terms of contaminant reduction, operating performance, and measured milk-quality changes. It was hypothesized that sequential combinations of sorbents would enhance contaminant reduction relative to single-sorbent treatments and that configuration selection would depend on contaminant type and the balance among removal efficiency, operational requirements, and changes in the measured milk-quality parameters.

## 2. Materials and Methods

### 2.1. Raw Cow Milk and Experimental Materials

Raw cow milk from Kazakh Whiteheaded cows was obtained from rural locations in Abay Region, Kazakhstan, with the milk source selected according to the purpose of each experimental component. Borodulikha milk was used to prepare Pb- and Cd-fortified model milk. Milk from Kainar and Sarzhal, which contained measurable native activities of ^137^Cs and ^90^Sr, was used without artificial radionuclide spiking in separate radionuclide-removal series with nominal sorbent loadings of 100 and 150 cm^3^ per active column, respectively. Samples from the two locations were not pooled. Because milk source, initial radionuclide activity, and sorbent loading varied simultaneously, these series were treated as separate source-specific experimental conditions rather than as a controlled comparison of sorbent loading. Unspiked Turksib milk was used separately to evaluate changes in physicochemical properties, viscosity, and Na, K, and Ca concentrations after filtration. The Turksib milk was not analysed for Pb, Cd, ^137^Cs, or ^90^Sr; therefore, its contaminant status and comparability with the milk used in the contaminant-removal experiments were not established. Accordingly, the contaminant-removal and milk-quality experiments represent complementary assessments conducted using different milk matrices and do not demonstrate contaminant reduction and preservation of the measured milk-quality parameters simultaneously in the same samples.

For the Pb/Cd experiments, three separate 10-L fortified batches were prepared for each loading series, giving six batches in total. Different milk batches were used for the 100 and 150 cm^3^ series.

Sampling for the Pb/Cd and physicochemical experiments was conducted on 3–10 July 2025, whereas radionuclide samples were collected on 10–15 July 2025. Milk was used raw and non-pasteurized. Immediately after milking, samples were cooled and transported in portable refrigerated containers at 2–4 °C. The maximum interval between milking and filtration was approximately 12–14 h; after laboratory arrival, samples were stored at 4–6 °C for no longer than 2–3 h. Before filtration, milk was manually mixed for 1–2 min, equilibrated to 18–20 °C, and remixed to ensure homogeneity.

For the physicochemical assessment, three individual-cow milk batches were used in Series A (100 cm^3^ per active column), and three different individual-cow batches were used in Series B (150 cm^3^ per active column). The two series were conducted on separate collection dates. Therefore, comparisons between Series A and Series B do not isolate the effect of sorbent loading from possible batch- and collection-date-related differences.

Before filtration, the mean initial values for Series A were 3.31% fat, 4.50% lactose, 11.56% total solids, 8.25% solids-not-fat, and 3.05% protein, with a density of 1028.32 kg/m^3^, a freezing point of −0.529 °C, and a pH of 6.62. The corresponding mean values for Series B were 3.28%, 4.46%, 11.46%, 8.18%, 3.03%, 1028.10 kg/m^3^, −0.525 °C, and 6.51, respectively. No added water was detected, and the milk used in both series complied with GOST 31449-2013 [25].

### 2.2. Sorbents and Their Pretreatment

Three granular sorbents were used: taurite (grade TK; LLP “GRK Koksu”, Almaty Region, Kazakhstan), natural zeolite (ST 72-1907-17-LLP-01-2020 [26]; LLP “Taza Su”, Almaty Region, Kazakhstan), and coconut-shell activated carbon (grade 207C; “AKVADOKTOR”, Ust-Kamenogorsk, Kazakhstan). According to product documentation, the specific surface areas of zeolite and activated carbon were approximately 420 and 1050–1150 m^2^/g, respectively; the specific pore volume of zeolite was 0.63 cm^3^/g.

The initial particle sizes were approximately 5 mm for taurite, 10–15 mm for zeolite, and 2–3 mm for activated carbon. Before use, the sorbents were crushed as required and sieved to obtain a 1–2 mm working fraction.

Nominal sorbent loadings of 100 and 150 cm^3^ per active column were evaluated. For 100 cm^3^, the dry masses were 136.44 g taurite, 136.15 g zeolite, and 82.81 g activated carbon; for 150 cm^3^, the corresponding masses were 206.62, 188.97, and 117.14 g. The resulting bed heights were 7.2, 8.9, and 7.8 cm for the 100-cm^3^ loading and 12.2, 13.0, and 11.9 cm for the 150-cm^3^ loading, respectively.

Before column packing, the sorbents were washed with distilled water and subjected to acid and alkaline pretreatment. Taurite and activated carbon were treated with 0.05–0.10 M HNO_3_, while zeolite was treated with 0.1 M HCl or HNO_3_; all sorbents were subsequently treated with 0.1 M NaOH and rinsed to approximately neutral pH. Taurite and zeolite were dried at 110–150 °C for 3–4 h, and activated carbon at 105–120 °C for 3–5 h, until constant mass. Prepared sorbents were stored in sealed glass containers at room temperature and protected from light. Fresh sorbent was used for each independent filtration run; regeneration and reuse were not investigated.

### 2.3. Design of the Adsorption–Filtration System

A laboratory-scale modular multistage adsorption–filtration unit, developed according to the patented design described in [22], was used to remove heavy metals and radionuclides from cow milk. The system comprised a support frame, a feed reservoir with an approximate capacity of 4–5 L, three vertical sorption columns, a centrifugal-impeller pump, control valves, a manometer, a flowmeter, and two collection vessels. The components were connected by polypropylene pipelines with an outer diameter of 20 mm. The assembled unit measured 140 × 200 × 230 mm and had a mass of 23 kg (Figure 1).

Each sorption column consisted of a dismountable polypropylene housing with upper and lower covers. The internal diameter was 38 mm, the cylindrical body length was 197 mm, and the total assembled length was 257 mm. Heat-, frost-, acid-, and alkali-resistant rubber gaskets ensured leak-tight operation. A food-grade retaining mesh with 0.5–1.0 mm apertures was installed at the bottom of each column to support the sorbent and prevent particle carryover. No upper retaining mesh, supporting layer, or distributor plate was used. The maximum sorbent-bed height was 22.7 cm (Figure 2).

Column 1 contained taurite, column 2 natural zeolite, and column 3 coconut-shell activated carbon 207C. Sorbents were loaded volumetrically at nominal volumes of 100 or 150 cm^3^ per active column before packing. These values refer to the volume of sorbent loaded into each active column and not to the volume of milk processed. Accordingly, the total nominal sorbent volume was 100 or 150 cm^3^ for single-column configurations, 200 or 300 cm^3^ for two-column configurations, and 300 or 450 cm^3^ for the three-column configuration. The corresponding bed heights for taurite, natural zeolite, and activated carbon 207C were 7.2, 8.9, and 7.8 cm, respectively, at the 100 cm^3^ loading and 12.2, 13.0, and 11.9 cm, respectively, at the 150 cm^3^ loading. Milk flowed downward through the sorbents under fixed-bed conditions. Approximately 1 L of filtrate was collected from each filtration configuration, and the first approximately 100 mL of filtrate was collected separately and excluded from subsequent analyses.

The unit permitted gravity-driven and forced-flow operation. In forced-flow mode, milk was delivered using a centrifugal-impeller pump (HYDROJET 32-80-180, Hydrojet; supplied by LLP “Avtomaticheskie Kotly”, Karaganda, Kazakhstan with nominal power settings of 135, 200, and 245 W. Preliminary water tests conducted at the three pump settings informed the selection of the second setting (nominally 200 W) for the principal milk-filtration experiments. The ZEIN manometer, installed in the supply line upstream of the column-distribution system, indicated an inlet gauge pressure of approximately 0.10 MPa (1 bar). Outlet pressure was not measured; therefore, the pressure differential across the packed bed was not determined. A GERRIDA SVK-15G water meter (LLC PKF “GERRIDA”, Krasnodar, Russia) was installed to monitor the flow. However, validated volumetric flow-rate data were not obtained for the milk-filtration runs. Consequently, the reported collection times should not be interpreted as direct measurements of hydraulic residence time or used to establish an optimum hydraulic loading.

The valve manifold enabled single-column operation (T, Z, AC), two-stage sequential configurations (T→Z, T→AC, Z→AC), and the three-stage sequence T→Z→AC. Although the unit could technically support parallel connections, only single-column and sequential configurations were used experimentally (Figure 3).

Before and between experiments, the system was repeatedly rinsed with bidistilled water; approximately 20–25 L of bidistilled water was used for the final rinse. The first approximately 1 L fraction passing through the system was discarded before analytical filtrate collection.

### 2.4. Experimental Design

The study comprised two experimental blocks: (i) removal of Pb and Cd from artificially contaminated cow milk and (ii) removal of naturally occurring ^137^Cs and ^90^Sr from native cow milk. In each block, an unfiltered control and seven filtration configurations were evaluated: taurite (T), zeolite (Z), activated carbon (AC), T→Z, T→AC, Z→AC, and T→Z→AC.

Two nominal sorbent loading volumes, 100 and 150 cm^3^ per active column, were tested. These values represented the bulk sorbent volume measured before packing. Accordingly, total nominal sorbent loadings were 100 or 150 cm^3^ for single-column treatments, 200 or 300 cm^3^ for two-column treatments, and 300 or 450 cm^3^ for the three-column treatment. The 100- and 150-cm^3^ experiments used separate milk batches, each with its own untreated control.

For the heavy-metal block, approximately 10 L of raw milk from Borodulikha was fortified with Pb and Cd. Working Pb and Cd solutions prepared from certified reference materials were added sequentially to the milk (Pb→Cd) under mechanical mixing. The measured pre-filtration concentrations were 0.36 mg/kg Pb and 0.32 mg/kg Cd for the 100-cm^3^ series and 0.34 mg/kg Pb and 0.31 mg/kg Cd for the 150-cm^3^ series. These analytically determined values were used as the initial concentrations for removal calculations.

For the radionuclide block, native milk from Kainar and Sarzhal was evaluated separately without artificial enrichment. Three independent samples were analyzed from each location. Initial activities were 1.20 Bq/kg ^137^Cs and 0.9981 Bq/kg ^90^Sr for the 100-cm^3^ series and 1.044 and 0.4314 Bq/kg, respectively, for the 150-cm^3^ series.

Each filtration configuration was performed in three independent runs (*n* = 3) using fresh sorbent. Each filtrate and corresponding control was analyzed in triplicate. Milk was mixed before filtration, equilibrated to 18–20 °C, and processed at approximately 1 bar. Treatments were conducted in the fixed order T, Z, AC, T→Z, Z→AC, T→AC, and T→Z→AC.

Primary responses were residual Pb, Cd, ^137^Cs, and ^90^Sr and their removal efficiencies. Secondary responses included physicochemical and quality characteristics of milk before and after filtration.

### 2.5. Preparation of Pb- and Cd-Spiked Milk

For the Pb/Cd experiments, three separate 10-L milk batches were prepared for each sorbent-loading series, giving six batches in total. Milk pooled from several cows in Borodulikha, Abay Region, Kazakhstan, was used, with different batches assigned to the 100 and 150 cm^3^ series. Each batch was fortified separately with Pb and Cd using certified reference solutions containing 1.0 g/dm^3^ of the respective element in dilute nitric acid. After fortification, the milk was mechanically mixed for approximately 5 min and divided into eight aliquots allocated to the unfiltered control and the seven column configurations. Filtration began approximately 1–1.5 h after fortification. Each independently prepared batch was considered an experimental unit; therefore, the control and filtration treatments obtained from the same batch shared a common starting milk matrix. Metal speciation and distribution among the different components of the milk matrix during the equilibration period were not determined.

Lead was introduced using a certified reference material for Pb ions (NK-EK, GSO 7877-2000; EKROSKHIM LLC, St. Petersburg, Russia; batch No. 01-24; production date 11 April 2024). Cadmium was introduced using a certified reference material for Cd ions (set No. 1K, GSO 6690-93; CSOVV LLC, St. Petersburg, Russia; batch No. 21/1K-1-CSO; production date 8 July 2022).

Working solutions were prepared separately for each element by diluting 1.0 mL of the corresponding certified reference solution to 100 mL with deionized water, followed by manual mixing until homogeneous. The Pb working solution was added first, followed by the Cd working solution (Pb→Cd), while the milk was mechanically mixed to promote uniform distribution.

The exact volumes of the Pb and Cd working solutions added to the 10-L milk batches were not recorded in the laboratory log. Therefore, the analytically determined concentrations immediately before filtration were used as the initial concentrations (C_0_). For the 100-cm^3^ sorbent-loading series, C_0_ values were 0.36 mg/kg Pb and 0.32 mg/kg Cd; for the 150-cm^3^ series, they were 0.34 and 0.31 mg/kg, respectively. These measured values were used to calculate Pb and Cd removal efficiencies.

After fortification, the milk was mechanically mixed to ensure homogeneous metal distribution and divided into portions for filtration and control treatments. Controls included native unspiked milk, spiked unfiltered milk, unspiked milk passed through prewashed sorbents, and a reagent blank. Empty-column and acid-matched controls were not included. Removal efficiency was calculated relative to the corresponding spiked unfiltered control.

### 2.6. Radionuclide-Containing Milk Samples

Milk from Kainar and Sarzhal was evaluated in separate radionuclide-filtration experiments. Three independent samples (*n* = 3) were collected from each location between 10 and 15 July 2025. Each sample represented milk from a single cow and had a volume of approximately 2–3 L. Before subsampling, the milk was mixed manually to ensure homogeneity. Samples were cooled immediately after collection and transported to the laboratory in portable refrigerated units at 2–4 °C within approximately 1 h. If analysis was not initiated immediately, samples were stored at 4–6 °C for no longer than 2–3 h.

Initial radionuclide activities were determined before adsorption–filtration. Kainar milk, used in the 100 cm^3^ series, contained 1.20 Bq/kg of ^137^Cs and 0.9981 Bq/kg of ^90^Sr. Sarzhal milk, used independently in the 150 cm^3^ series, contained 1.044 Bq/kg of ^137^Cs and 0.4314 Bq/kg of ^90^Sr. Because milk origin and sorbent loading varied simultaneously, the two series were evaluated separately and were not used to estimate the independent effect of sorbent-bed volume.

Determination of ^137^Cs and ^90^Sr was performed at the Testing Center, Semey Branch of JSC “National Center for Expertise and Certification”. ^137^Cs was measured by gamma-ray spectrometric analysis, whereas ^90^Sr was determined after separation from the milk matrix using the accredited procedures of the testing laboratory.

The measured initial activities were used as baseline values for calculating radionuclide-removal efficiency after filtration.

### 2.7. Filtration Procedure

Filtration experiments were performed using the modular adsorption–filtration unit described in Section 2.3. The same general procedure was applied to Pb/Cd-spiked milk and native milk containing ^137^Cs and ^90^Sr.

Before the first experiment and between independent runs, all milk-contact surfaces were repeatedly rinsed with bidistilled water; approximately 20–25 L was used for final rinsing. The required column configuration was then assembled and packed with fresh sorbent corresponding to nominal loadings of 100 or 150 cm^3^ per active column. Sorbent masses and bed heights are given in Section 2.2. Fresh sorbent was used for every independent run.

Immediately before filtration, milk was manually mixed for approximately 1–2 min, equilibrated to 18–20 °C, and an unfiltered control was collected. The valves were adjusted to direct milk through the selected single- or multicolumn configuration. Filtration was performed under forced flow using the centrifugal-impeller pump described in Section 2.3, at an operating pressure of approximately 1.0 bar. Filtration was performed under forced flow using the centrifugal-impeller pump described in Section 2.3, at an operating pressure of approximately 1.0 bar. The reported filtration times refer to the collection of approximately 1 L of filtrate. Timing began with the first drop at the outlet and ended with the last drop. Configuration-specific collection times ranged from 10 to 22 s. These collection times are not direct measurements of fluid residence time within the sorbent bed.

The first approximately 1 L fraction was discarded. The main filtrate volume was approximately 1.0–1.5 L per configuration and was mixed manually before analytical subsampling. Approximately 50 mL was collected for Pb and Cd determination, 1 L for radionuclide analysis, and 100–200 mL for physicochemical measurements. Samples were stored at 4–6 °C and analyzed within approximately 2–3 h.

Each treatment was performed in three independent filtration runs (*n* = 3), and each filtrate was analyzed in triplicate. After each run, residual milk was drained, spent sorbent was removed, and the system was repeatedly rinsed with bidistilled water before repacking. Pb/Cd experiments were completed before the radionuclide experiments to minimize cross-contamination between experimental blocks.

### 2.8. Determination of Pb and Cd in Milk Samples

Pb and Cd concentrations in milk samples were determined using an Agilent 240FS AA atomic absorption spectrometer (Agilent Technologies, Santa Clara, CA, USA). Samples were prepared and analyzed using standard procedures for Pb and Cd determination [27]. Certified reference solutions of Pb and Cd were used for preparation of the fortified milk samples and analytical control. Measurements were conducted at 23–25 °C, 61–64% relative humidity, and an atmospheric pressure of approximately 740 mmHg. Pb and Cd concentrations were expressed in mg/kg of milk.

### 2.9. Determination of ^137^Cs and ^90^Sr in Milk Samples

^137^Cs activity was determined by gamma-ray spectrometry in accordance with GOST 32161-2013 [28], using a GC-2019 pure germanium detector (55 cm^3^; Canberra Industries, Inc., Meriden, CT, USA), following the analytical approach previously applied for radionuclide determination in cow milk. Milk samples were transferred to 1-L Marinelli beakers (STC RADEK Ltd., St. Petersburg, Russia) and placed directly on the detector for gamma-spectrometric measurement. The spectrometer was calibrated using certified gamma-emitting reference sources, including ^152^Eu, ^241^Am, and ^137^Cs. The detection limit for this gamma-spectrometric system was 0.1 Bq for a 0.5-kg sample.

^90^Sr activity was determined by beta spectrometry using a Progress-Beta-M beta spectrometer (“Dosa” Research and Production Enterprise, Moscow, Russia) in accordance with GOST 32163-2013 [29]. Milk samples were prepared as counting samples prior to beta-spectrometric measurement. When the concentration of the radionuclide was required, sample preparation was performed according to the prescribed concentration procedures for milk. The minimum measurable activity of the beta-spectrometric system was within the range of 0.1–1.0 Bq.

Measurements were conducted under laboratory conditions of 23–25 °C, 61–64% relative humidity, and an atmospheric pressure of approximately 740 mmHg. Radionuclide activities were expressed as Bq/kg of milk.

### 2.10. Determination of Fat Content

Milk fat content was determined by the acid butyrometric method according to GOST 5867-90 [30]. The method is based on the release of fat from milk proteins using concentrated sulfuric acid and isoamyl alcohol, followed by centrifugation and volumetric determination of the separated fat layer in a graduated butyrometer (PJSC “Steklopribor”, Zavodske, Poltava Region, Ukraine). Milk samples were thoroughly mixed before analysis. After addition of the prescribed volumes of sulfuric acid, milk, and isoamyl alcohol, the sealed butyrometers were mixed, incubated in a water bath at 65 ± 2 °C, and centrifuged according to the standard procedure. Fat content was read directly from the butyrometer scale and expressed as a percentage (%). Measurements were performed in duplicate, and the mean value was used for analysis.

### 2.11. Determination of Protein Content

Milk protein content was determined by the Kjeldahl method according to GOST 23327-98 [31]. The method involves digestion of the sample with concentrated sulfuric acid in the presence of a catalyst, followed by alkalization, ammonia distillation, and titration. Total nitrogen content was converted to protein using the milk-specific conversion factor of 6.38. A blank determination was performed in parallel. Protein content was expressed as a percentage (%), and the analysis was performed in duplicate with the mean value used for subsequent evaluation.

### 2.12. Determination of Lactose Content

Lactose content in raw and filtered milk samples was determined according to GOST 34304-2017 by an enzymatic spectrophotometric method [32]. Milk samples were thoroughly mixed, and proteins and fat were removed using zinc sulfate and potassium hexacyanoferrate solutions. The clarified filtrate was subjected to enzymatic hydrolysis of lactose with β-galactosidase, followed by oxidation of the resulting galactose in the presence of nicotinamide adenine dinucleotide (NAD) and galactose dehydrogenase.

The resulting reduced form, NADH, was measured spectrophotometrically at 340 nm, and lactose content was calculated from the corresponding change in absorbance. Results were expressed as mass fraction of lactose (%). Measurements were performed in duplicate, and the mean value was used for subsequent analysis.

### 2.13. Determination of Total Solids

Total solids in milk samples were determined according to GOST R 54668-2011 by oven drying [33]. A homogenized milk sample was weighed into a pre-dried weighing bottle containing prepared sand and a glass rod and dried at 102 ± 2 °C. Drying was continued to constant mass, defined as a difference of no more than 0.001 g between consecutive weighings. Total solids were calculated from the mass remaining after drying and expressed as a percentage (%). Moisture content was calculated as 100 − total solids. Measurements were performed in duplicate, and the mean value was used for subsequent analysis.

### 2.14. Determination of Solids-Not-Fat Content

The solids-not-fat (*SNF*) content of raw and filtered milk was determined according to GOST R 54761-2011 [34]. SNF was calculated as

*SNF* (%) = *Total solids* (%) − *Fat* (%)

### 2.15. Determination of Milk Density

The density of raw and filtered milk was determined by the aerometric method according to GOST R 54758-2011 using a milk hydrometer (PJSC “Steklopribor”, Zavodske, Poltava Region, Ukraine) [35]. Milk samples were thoroughly mixed, avoiding foam formation, and transferred into a dry measuring cylinder. Density was measured at 20 ± 5 °C after the hydrometer reached a stable floating position without contact with the cylinder walls. Two consecutive hydrometer readings were recorded, and their mean was calculated. When measurements were performed at temperatures other than 20 °C, the density values were corrected to the reference temperature of 20 °C using the correction tables specified in GOST R 54758-2011. Results were expressed as kg/m^3^.

### 2.16. Determination of pH

Milk pH was determined potentiometrically according to GOST R 53359-2009 using a pH-410 m (NPP Tom’Analit LLC, Tomsk, Russia) [36]. The instrument was calibrated with standard buffer solutions before measurement. Milk samples were equilibrated to 20 ± 2 °C, and pH was measured directly after stabilization of the electrode reading.

### 2.17. Determination of Dynamic Viscosity

The dynamic viscosity of raw and filtered milk samples was determined using a vibrational viscometer (Vibro Viscometer SV-10, A&D Company, Limited, Tokyo, Japan) with a measurement range of 0.3–10,000 mPa·s. Before analysis, milk samples were gently mixed to ensure homogeneity while minimizing air incorporation. For each measurement, 150–200 mL of milk was transferred to the measuring vessel and equilibrated to 25 °C. The sensor plates of the viscometer were immersed in the sample according to the manufacturer’s operating procedure, and viscosity was recorded after the reading had stabilized. No spindle was used because the SV-10 operates on the vibration-based measurement principle. Each sample was measured three times, and the results were expressed as dynamic viscosity in mPa·s.

### 2.18. Determination of Mineral Composition

The mineral composition of raw and filtered milk samples was determined by inductively coupled plasma mass spectrometry (ICP-MS) using a Varian 820-MS instrument (Varian Inc., Melbourne, Australia). Before analysis, milk samples were homogenized and subjected to acid digestion using concentrated nitric acid under controlled heating until complete mineralization was achieved. The digested samples were then diluted with ultrapure water prior to instrumental analysis.

The concentrations of sodium (Na), potassium (K), and calcium (Ca) were quantified using certified multi-element calibration standards prepared in a matrix-matched solution. Calibration curves were constructed using at least five concentration levels covering the expected range of analytes, and linearity was verified prior to sample measurement. Instrument calibration was performed at the beginning of each analytical sequence, and calibration verification standards were analyzed periodically to ensure instrumental stability. Analytical blanks were included in each batch to monitor potential contamination and background signal.

ICP-MS measurements were carried out under standard operating conditions recommended for the Varian 820-MS system, including optimization of plasma conditions, ion lens settings, and mass calibration prior to analysis. Data acquisition was performed in triplicate for each sample, and results were corrected for blank values. Mineral concentrations were expressed as mg/kg of milk. All samples were analyzed in triplicate, and mean values were used for statistical comparison between untreated and filtered milk samples [37].

### 2.19. Statistical Analysis

For the Pb/Cd experiments, three independent 10-L milk batches were prepared for each sorbent-loading series (*n* = 3 batches per series). Each batch was divided among the unfiltered control and the seven filtration configurations (T, Z, AC, T→Z, T→AC, Z→AC, and T→Z→AC). Thus, the milk batch was considered the experimental replicate, while the control and filtration treatments obtained from the same batch were treated as related observations. For the physicochemical assessment, three individual-cow milk batches were used for each loading series (*n* = 3). Different milk batches were used for the 100 and 150 cm^3^ series. Analytical measurements were performed in triplicate for each sample. These measurements were treated as technical replicates, and their mean was used as the value for the corresponding experimental run. Technical replicates were not treated as independent observations.

Data are presented as mean ± standard deviation (SD), calculated from the three independent experimental-run values (*n* = 3). Within each sorbent-loading series, differences among the unfiltered control and the seven filtration configurations (S, Z, AC, S→Z, S→AC, Z→AC, and S→Z→AC) were evaluated using randomized-block analysis of variance (ANOVA), with filtration configuration treated as the fixed factor and milk batch as the blocking factor. When ANOVA indicated a significant treatment effect, Tukey’s honestly significant difference (HSD) test was applied for multiple comparisons.

Radionuclide data were analysed separately for the Kainar/100 cm^3^ and Sarzhal/150 cm^3^ experimental series using one-way ANOVA, followed by Tukey’s HSD test where a significant configuration effect was detected. No cross-series ANOVA was performed because milk source, initial radionuclide activity, and sorbent loading varied simultaneously. Consequently, differences between the two series were interpreted descriptively and were not attributed independently to sorbent loading. Differences were considered statistically significant at *p* ≤ 0.05. Statistical analyses were performed using Microsoft Excel 2016 (Microsoft Corporation, Redmond, WA, USA) and IBM SPSS Statistics 29.0 (IBM Corp., Armonk, NY, USA).

## 3. Results

### 3.1. Sorbent Characterization

Table 1 summarizes the mineralogical, chemical, and surface-property information provided in the supplier quality documentation for the sorbent batches used in this study. According to these supplier-reported data, the three sorbents differed substantially in origin, composition, and physicochemical characteristics. Taurite TK was used as the standardized name for the taurite-derived commercial product. Before column loading, all sorbents were crushed and sieved, and the 1–2 mm particle-size fraction was confirmed by sieve analysis. Taurite TK from the Koksu deposit was a carbon–mineral material containing calcite, quartz, sericite, and carbonaceous matter, with 7–15% C and 30–55% SiO_2_. Its bulk density was approximately 0.60 g/cm^3^ and moisture content ≤ 1.0%.

Natural zeolite from the Chankanai deposit was predominantly clinoptilolite with quartz and feldspars. It contained 60–74% SiO_2_ and 14–15% Al_2_O_3_, had a bulk density of 1.17–1.32 g/cm^3^, a specific surface area of 420 m^2^/g, and a specific pore volume of 0.63 cm^3^/g. Activated carbon 207C, produced from coconut shells, contained 80–90% carbon and had a bulk density of 0.49–0.53 g/cm^3^, moisture content ≤ 5%, and the highest reported specific surface area, 1050–1150 m^2^/g. Thus, the sorbents represented a carbon–mineral composite, a clinoptilolite-rich aluminosilicate, and a highly porous carbonaceous adsorbent, respectively.

### 3.2. Removal of Pb and Cd

During the principal experiments, the upstream manometer indicated an inlet gauge pressure of approximately 0.10 MPa (1 bar). Milk flow appeared stable during the short filtration runs, with no visible channel formation, sorbent-bed displacement, compaction, or preferential wall flow. However, configuration-specific outlet pressure and pressure-drop data were not available; therefore, the hydraulic resistance of the packed beds could not be quantified. Under these operating conditions, Pb and Cd concentrations decreased to different extents depending on the column configuration and experimental series (Figure 4).

The initial Pb concentrations were 0.360 and 0.340 mg/kg in the independently prepared 100 and 150 cm^3^ series, respectively. In the 100 cm^3^ series, Pb concentration decreased to 0.033 mg/kg after complete three-stage filtration, corresponding to a removal efficiency of 90.83%. In the 150 cm^3^ series, it decreased to 0.018 mg/kg, corresponding to 94.71% removal. Within each series, Pb concentrations differed significantly among the column configurations (*p* < 0.05).

A similar pattern was observed for Cd. The initial concentrations were 0.320 and 0.310 mg/kg in the 100 and 150 cm^3^ series, respectively. Complete three-stage filtration reduced Cd concentration to 0.027 mg/kg in the 100 cm^3^ series and 0.012 mg/kg in the 150 cm^3^ series, corresponding to removal efficiencies of 91.56% and 96.13%, respectively. Within each series, Cd concentrations differed significantly among the column configurations (*p* < 0.05).

The maximum permissible concentrations in raw milk under TR CU 021/2011 [38] are 0.10 mg/kg for Pb and 0.03 mg/kg for Cd. The taurite→activated carbon and complete three-stage configurations reduced both metals below these limits in both experimental series. The complete three-stage configuration produced Pb and Cd removal efficiencies that were numerically 3.88 and 4.57 percentage points higher, respectively, in the 150 cm^3^ series than in the 100 cm^3^ series. However, because different independently prepared milk batches were used, these differences cannot be attributed exclusively to sorbent loading.

The reported removal efficiencies represent decreases in Pb and Cd concentrations in the recovered milk rather than direct measurements of the total metal mass retained by the system. Because complete milk-volume and contaminant mass balances were not established and the spent sorbents were not analysed, the amounts and chemical forms of Pb and Cd retained within the beds could not be determined.

### 3.3. Removal of ^137^Cs and ^90^Sr

Following the Pb and Cd experiments, the sorbent-based filtration unit was evaluated using cow milk containing pre-existing, non-spiked ^137^Cs and ^90^Sr. Kainar milk, used in the 100 cm^3^-per-layer series, had initial activities of 1.20 Bq/kg for ^137^Cs and 0.9981 Bq/kg for ^90^Sr. Sarzhal milk, used in the independent 150 cm^3^-per-layer series, had corresponding initial activities of 1.044 and 0.4314 Bq/kg. Because milk source, initial radionuclide activity, milk-matrix characteristics, and sorbent loading differed simultaneously, the two series were evaluated separately. All initial and post-filtration activities were substantially below the regulatory limits of 100 Bq/kg for ^137^Cs and 25 Bq/kg for ^90^Sr.

In the Kainar/100 cm^3^ series, single-sorbent filtration reduced ^137^Cs activity to 0.600–1.044 Bq/kg and ^90^Sr activity to 0.548–0.898 Bq/kg. The complete taurite TK→natural zeolite→activated carbon 207C configuration reduced ^137^Cs to 0.383 Bq/kg and ^90^Sr to 0.346 Bq/kg, corresponding to removal efficiencies of approximately 68.1% and 65.3%, respectively. Radionuclide activities differed significantly among the column configurations within this series (*p* < 0.05).

In the Sarzhal/150 cm^3^ series, the complete three-stage configuration reduced ^137^Cs activity from 1.044 to 0.210 Bq/kg and ^90^Sr activity from 0.4314 to approximately 0.096 Bq/kg, corresponding to removal efficiencies of approximately 79.9% and 77.7%, respectively. Radionuclide activities also differed significantly among the column configurations within this series (*p* < 0.05).

Across both source-specific series, multistage configurations containing natural zeolite generally produced lower residual radionuclide activities than the single-sorbent configurations, while the complete three-stage system yielded the lowest residual activities within each series (Figure 5). No inferential comparisons were performed between the Kainar/100 cm^3^ and Sarzhal/150 cm^3^ series. Consequently, the numerical differences between them cannot be attributed solely to sorbent-bed volume. Establishing an independent loading effect would require aliquots of the same homogenized milk batch to be treated at both loading levels under otherwise identical operating conditions.

### 3.4. Effects of Adsorption–Filtration on Milk Quality

The physicochemical characteristics of milk were evaluated before and after adsorption–filtration using sorbent loadings of 100 and 150 cm^3^ per layer. For multistage configurations, the stated loading refers to each individual sorbent bed.

#### 3.4.1. Changes in Macronutrient Composition and pH

The effects of adsorption–filtration on milk composition depended on the sorbent configuration (Table 2). Within both experimental series, taurite, activated carbon 207C, and particularly the taurite→activated carbon configuration produced relatively small changes compared with untreated milk. In contrast, natural zeolite and several zeolite-containing configurations resulted in significantly higher measured fat concentrations (*p* < 0.05). No significant differences in lactose concentration were observed among column configurations within either series (*p* > 0.05).

For the taurite→activated carbon system, fat, lactose, protein, total solids, and SNF changed from 3.31, 4.50, 3.05, 11.56, and 8.25% to 3.29, 4.49, 3.02, 11.32, and 8.21%, respectively, in Series A, and from 3.28, 4.46, 3.03, 11.46, and 8.18% to 3.24, 4.44, 3.02, 11.28, and 8.16% in Series B. These changes were generally not significant relative to untreated milk (*p* > 0.05). By comparison, the complete three-stage system produced greater numerical increases in fat, protein, total solids, and SNF, although statistical significance varied among parameters. In particular, differences in fat were significant (*p* < 0.05), whereas SNF remained statistically unchanged (*p* > 0.05).

Milk density varied only slightly, from 1028.25 to 1029.70 kg m^−3^ in Series A and from 1028.00 to 1029.52 kg m^−3^ in Series B, with no significant differences among configurations (*p* > 0.05). The pH also decreased numerically after filtration but did not differ significantly among configurations within either series (*p* > 0.05). For the taurite→activated carbon system, pH changed from 6.62 to 6.52 in Series A and from 6.51 to 6.41 in Series B. The freezing point remained unchanged within each series, and no added water was detected.

No significant differences were observed between Series A and Series B for the corresponding basic physicochemical parameters listed in Table 2 (*p* > 0.05). Apparent increases in fat, protein, and total solids should nevertheless be interpreted cautiously because partial retention of the liquid phase within the sorbent beds may concentrate the remaining milk. Overall, the taurite→activated carbon configuration produced the smallest deviations from the initial milk composition among the high-efficiency treatment systems.

#### 3.4.2. Changes in Dynamic Viscosity After Adsorption–Filtration

Dynamic viscosity, measured at 25 °C, decreased slightly after filtration in all column configurations (Figure 6). In Series A, viscosity decreased from 1.99 mPa·s in untreated milk to 1.90–1.95 mPa·s after filtration, with the lowest value observed for the complete taurite→zeolite→activated carbon system. This corresponded to a maximum reduction of 4.52%. However, the differences among column configurations within Series A were not statistically significant (*p* > 0.05).

A similar trend was observed in Series B, where viscosity decreased from 1.38 mPa·s in untreated milk to 1.31–1.35 mPa·s after filtration. The complete three-stage configuration again produced the lowest value, corresponding to a 5.07% reduction. As in Series A, differences among column configurations within Series B were not significant (*p* > 0.05).

In contrast, viscosity values differed significantly between Series A and Series B for each corresponding column configuration (*p* < 0.05). Overall, adsorption–filtration caused only modest, statistically non-significant changes in milk viscosity within each experimental series.

#### 3.4.3. Changes in Mineral Composition After Adsorption–Filtration

Adsorption–filtration affected the concentrations of Na, K, and Ca to different extents depending on the sorbent configuration (Figure 7). The smallest changes were generally observed after filtration through taurite, activated carbon 207C, and their two-stage combination, whereas configurations containing natural zeolite produced more pronounced reductions, particularly in Na and K.

In Series A, the taurite→activated carbon 207C configuration changed Na from 1839 to 1837.3 mg/L, K from 1564 to 1561 mg/L, and Ca from 600 to 598 mg/L. In Series B, the corresponding values changed from 1011.56 to 1010.01 mg/L for Na, from 1407.6 to 1404.68 mg/L for K, and from 400 to 398 mg/L for Ca. For this configuration, the mineral concentrations did not differ significantly from the corresponding untreated milk within each experimental series (*p* > 0.05).

In contrast, natural zeolite alone and several zeolite-containing multistage systems caused significantly greater reductions in Na and K within the same experimental series (*p* < 0.05). After complete three-stage filtration, Na decreased from 1839 to 1700 mg/L and K from 1564 to 1370 mg/L in Series A, while in Series B Na decreased from 1011.56 to 936 mg/L and K from 1407.6 to 1239 mg/L. Calcium was less affected, and no significant differences among column configurations were observed within either series (*p* > 0.05).

Concentrations of all three minerals differed significantly between Series A and Series B within the corresponding column configurations (*p* < 0.05). However, because different milk batches collected on different dates were used for the two series, these differences cannot be attributed solely to sorbent loading. Within each series, configurations containing natural zeolite generally produced the greatest decreases in measured Na and K concentrations, whereas the taurite→activated carbon 207C configuration produced the smallest changes. These values represent net concentrations in the filtered milk and do not constitute an elemental mass balance.

### 3.5. Comparative Evaluation of Column Configurations

The column configurations were compared at a fixed sorbent loading of 150 cm^3^ per layer using Pb and Cd removal, regulatory compliance, ^137^Cs and ^90^Sr removal, preservation of the Na, K, and Ca profile, and filtration time as comparative criteria (Table 3). The maximum permissible concentrations for raw milk were 0.10 mg/kg for Pb and 0.03 mg/kg for Cd.

Among the single-sorbent configurations, activated carbon 207C showed the highest mean Pb/Cd removal efficiency (64.61%), although the residual concentrations of both metals remained above the regulatory limits. The two-stage taurite→natural zeolite and natural zeolite→activated carbon systems reduced Pb below the permissible concentration but did not achieve compliance for Cd.

Only the taurite→activated carbon 207C (1 + 3) and complete taurite→natural zeolite→activated carbon 207C (1 + 2 + 3) configurations reduced both Pb and Cd below the regulatory limits. The 1 + 3 system reduced Pb and Cd to 0.032 and 0.025 mg/kg, respectively, corresponding to a mean Pb/Cd removal efficiency of 91.26%. This configuration also showed the highest mean retention of the measured minerals (99.71%) and required 15 s of filtration.

The complete three-stage system provided the highest mean Pb/Cd removal efficiency (95.42%), reducing Pb and Cd to 0.018 and 0.012 mg/kg, respectively. It also produced the greatest radionuclide removal, reaching 79.87% for ^137^Cs and 77.68% for 90Sr. However, mean mineral retention decreased to 92.93%, mainly because Na and K decreased more, and filtration time increased to 22 s.

Radionuclide performance differed somewhat from that observed for Pb and Cd. The natural zeolite→activated carbon 207C configuration showed higher ^137^Cs and ^90^Sr removal than the taurite→activated carbon system, indicating that the optimal configuration depended on the contaminant targeted. Nevertheless, the complete three-stage system provided the highest overall decontamination performance across Pb, Cd, ^137^Cs, and ^90^Sr.

Considering regulatory compliance for Pb and Cd, radionuclide removal, preservation of the mineral profile, and filtration time, the taurite→activated carbon 207C configuration was identified as the best-balanced treatment for heavy-metal removal with minimal alteration of milk composition, whereas the complete three-stage configuration was the most effective when maximum simultaneous removal of heavy metals and radionuclides was prioritized.

All configurations operated at approximately 1 bar (0.10 MPa). Configuration-specific pressure-drop data were not available and were therefore not included in the comparative evaluation.

### 3.6. Practical Limitations and Scale-Up

The study demonstrated the feasibility of reducing Pb and Cd concentrations in milk using sequential sorbent beds; however, several parameters relevant to practical implementation remain unresolved. Sorbent service life and breakthrough behavior were not determined because each bed was used only once. Regeneration of taurite, natural zeolite, and activated carbon 207C was not evaluated, and the safety of reusing regenerated sorbents remains unknown. Therefore, the sorbents should currently be considered single-use materials.

The Pb and Cd contents of spent sorbents and their leaching behavior were not assessed, so an appropriate disposal procedure has yet to be established. After filtration, the columns were rinsed with approximately 20–25 L of bidistilled water without detergents or disinfectants. Removal of residual milk components and microbiological cleanliness were not verified; therefore, a validated sanitation protocol is required before repeated food-processing use.

The present study evaluated laboratory-scale column filtration. Sustained throughput and continuous-operation performance were not systematically validated. Further pilot-scale work should assess breakthrough, fouling, flow stability, contaminant reduction during prolonged operation, sanitation and changes in the measured milk-quality parameters.

## 4. Discussion

The present study demonstrates configuration-dependent reductions in Pb, Cd, ^137^Cs, and ^90^Sr in cow milk by sequential sorbent-based filtration. Coconut-shell activated carbon was the most effective single sorbent, while multistage configurations enhanced removal. Taurite→activated carbon (T→AC) achieved 90.59% Pb and 91.93% Cd reduction in the 150 cm^3^-per-layer series. When considered alongside the separately measured milk-quality results, this configuration offered a favourable balance among the evaluated criteria, rather than demonstrating simultaneous decontamination and quality preservation in the same milk. Zeolite-containing configurations were advantageous for radionuclide reduction, whereas the complete three-stage system required more sorbent. Configuration selection therefore depends on the target contaminant and the measured quality and operational criteria.

The Pb/Cd results are consistent with earlier milk-decontamination studies. Abdelnaby et al. (2023) reported Pb/Cd removal efficiencies of 84/88% with bentonite, 97/93% with date-pit powder, and 82/98% with chitosan nanoparticles [39]. Massoud et al. (2020) obtained 80% Pb and 75% Cd removal using *Lactobacillus acidophilus*, although treatment required high bacterial biomass and several days of contact [40]. Shen et al. (2018) achieved approximately 85% Pb removal from whole milk using a P123-DA hydrogel [41]. These findings support the feasibility of metal reduction in milk, but differences in sorbents, initial concentrations, contact times, and treatment modes preclude direct performance rankings. The present work applies sequential inorganic and carbonaceous beds in a flow-through configuration.

The greater Pb/Cd reduction with S→AC than with either single bed suggests complementary retention processes. Activated-carbon surface sites and the mineral–carbon character of taurite may provide different binding environments. However, configuration rankings alone cannot distinguish complementary surface interactions from the effects of additional bed volume or other retention processes. Ge et al. (2021) demonstrated antibiotic adsorption from milk by activated carbon despite competition from milk constituents and linked adsorption to oxygen-containing surface groups [42]. Their granular-carbon column results support the feasibility of carbon treatment in a dairy matrix, but antibiotic removal does not directly establish the mechanism of metal retention.

The initial Pb (0.34–0.36 mg/kg) and Cd (0.31–0.32 mg/kg) concentrations were analytical challenge levels, approximately 3.4–3.6 and 10.3–10.7 times their respective regulatory limits, selected to permit measurement of concentration changes rather than reproduce typical raw-milk contamination. Soluble certified reference solutions in dilute nitric acid were added, followed by mixing, approximately 1–1.5 h before filtration. Metal speciation and equilibration with milk constituents were not verified. Naturally contaminated milk may differ in chemical forms and equilibration history. Removal may also depend on initial concentration, the sorbent-to-contaminant ratio, matrix competition, and analytical sensitivity at low residual levels. Validation near the regulatory limits and in naturally contaminated milk is therefore needed before extrapolating these efficiencies.

For radionuclides, the Kainar/100 cm^3^ and Sarzhal/150 cm^3^ series represent separate source-specific conditions. Within both series, multistage zeolite-containing treatments generally enhanced reduction. In Sarzhal milk, activated carbon alone removed 64.55% of ^137^Cs and 59.89% of ^90^Sr; zeolite→activated carbon (Z→AC) achieved 75.73% and 73.97%, and the three-stage system achieved 79.86% and 77.68%, respectively. Between-series differences cannot isolate loading effects because milk source, initial activity, and matrix characteristics varied simultaneously. A controlled loading comparison requires aliquots of the same homogenized milk batch at both bed volumes. The Pb/Cd loading series likewise used different independently prepared batches, so their numerical differences should not be attributed exclusively to bed volume.

The benefit of Z→AC is compatible with zeolitic cation exchange. Abusafa and Yücel (2002) demonstrated ^137^Cs retention by natural clinoptilolite under flow conditions and related sorption to its exchangeable cations [43]. Lim et al. (2024) reported high Sr^2+^ adsorption capacities for kaolin-derived Na-A zeolites, associated with greater crystallinity [44]. Ito et al. (2024) likewise demonstrated Sr^2+^ and Cs^+^ removal through exchange with framework-associated cations [45]. These aqueous-system studies support a possible mechanism, not direct quantitative comparisons with milk.

Matrix competition may help explain differences from aqueous systems. Izosimova et al. (2022) reported competition from Na^+^, Ca^2+^, Mg^2+^, and K^+^ during Cs/Sr sorption on aluminosilicate clays [46]. Lim et al. also found that coexisting Ca reduced Sr adsorption to some extent [44]. These observations are relevant to milk, which contains native mineral ions and constituents that can affect contaminant availability. They support contaminant-specific sorbent selection, while leaving the mechanisms in the present milk system unresolved.

The observed decreases are concentration-based outcomes in recovered milk, not measurements of total contaminant mass retained by the beds. Possible contributions include adsorption, ion exchange, precipitation, physical retention, and retention of contaminant-associated milk constituents. Pb and Cd may occur in soluble forms or associate with caseins, whey proteins, colloidal calcium phosphate, and other constituents; their reduction cannot be assigned solely to adsorption of free ions. The proposed surface interactions are therefore interpretations rather than experimentally established mechanisms. No pre- and post-filtration FTIR, XPS, or comparable surface analyses were performed to verify these interactions. Determining contaminant fate requires speciation and complete milk-recovery and contaminant mass balances covering filtered milk, retained solids, rinsing fractions, and spent sorbents.

Changes in measured milk-quality parameters are a second treatment-selection criterion. Taurite, activated carbon, and particularly S→AC produced relatively small physicochemical and viscosity changes, whereas zeolite-containing configurations caused more noticeable compositional changes. Viscosity remained close to the corresponding untreated milk within each batch. Abdelnaby et al. (2023) similarly reported no substantial fat, protein, or lactose changes after their treatments [39], while Shen et al. (2018) reported protein and lactose preservation and minor Ca and Mg losses [41]. Such findings support evaluating measured quality alongside contaminant reduction, rather than selecting treatments on removal efficiency alone.

Zeolite-containing configurations produced larger Na and K decreases than taurite or activated carbon, whereas Ca was less affected. This is compatible with the exchangeable cations of aluminosilicates described by Șenilă et al. (2023) [47] and the competitive interactions reported by Izosimova et al. [46]. Nevertheless, only inlet and filtered-milk Na, K, and Ca were quantified. Without analyses of retained milk, rinsing water, time-resolved effluent, and spent sorbents, the data cannot distinguish exchange or adsorption from physical retention, soluble–colloidal redistribution, concentration effects, or simultaneous elemental uptake and release. These are net concentration changes, not direct evidence of mineral exchange. The restricted elemental panel also prevents conclusions about preservation of the complete mineral profile; broader mineral analysis and quantitative leaching assessment are required.

Contaminant reduction and quality changes were assessed in different milk matrices. Turksib milk, used for physicochemical, viscosity, and mineral analyses, was not analysed for Pb, Cd, ^137^Cs, or ^90^Sr. Consequently, simultaneous contaminant reduction and maintenance of measured quality in the same contaminated samples remain unconfirmed. Future validation should measure both before and after treatment of the same homogenized contaminated milk batches. Sensory acceptability also remains unestablished: qualitative observations do not replace a structured sensory assessment. Future sensory testing should use milk confirmed safe for panel evaluation.

At the nominal 200 W pump setting and approximately 1 bar inlet gauge pressure, reported collection times for approximately 1 L in the 150 cm^3^ series were 13 s for Z→AC, 15 s for S→AC, and 22 s for S→Z→AC. The three-stage configuration had the largest total bed volume (450 cm^3^) and longest reported collection time, suggesting a trade-off between contaminant reduction, sorbent use, and filtration rate. These short-run collection times do not measure bed residence time or establish continuous throughput. Pressure and flow were not varied systematically, and inlet gauge pressure does not represent a measured packed-bed pressure differential. Hydraulic loading, residence-time effects, and optimal operating conditions therefore remain undetermined.

Practical application requires further validation. Each bed was used only once; breakthrough behaviour, dynamic capacity, service life, and performance during repeated use remain unknown. Regeneration and spent-sorbent leaching were not assessed, so reuse and disposal require validated protocols. Rinsing with bidistilled water does not establish removal of milk residues or microbiological cleanliness. Longer-term trials should evaluate sanitation, stable flow, sorbent replacement or regeneration, and process economics alongside the contaminant-fate and quality assessments described above. The present study provides laboratory-scale evidence for selecting configurations according to contaminant type, but does not establish industrial readiness or comprehensive nutritional preservation.

## 5. Conclusions

The modular sorbent-based filtration system substantially reduced Pb and Cd concentrations in fortified Borodulikha milk and decreased native ^137^Cs and ^90^Sr activities under the separately tested Kainar/100 cm^3^ and Sarzhal/150 cm^3^ conditions. Among the evaluated configurations, taurite→activated carbon (T→AC) provided the best balance between Pb/Cd reduction and limited changes in the measured milk-quality parameters, whereas zeolite-containing multistage configurations achieved the greatest radionuclide reductions. These concentration-based results demonstrate contaminant reduction in the recovered milk but do not identify adsorption or any other individual process as the exclusive removal mechanism. The radionuclide results do not establish an independent sorbent-loading effect because milk source, initial activity, and sorbent loading varied simultaneously. Moreover, physicochemical properties were evaluated separately using Turksib milk; therefore, simultaneous contaminant reduction and preservation of milk-quality parameters in the same samples remain to be confirmed. Overall, the findings demonstrate laboratory-scale feasibility rather than industrial readiness. Further studies should establish contaminant fate through mass-balance, speciation, and spent-sorbent analyses and evaluate milk recovery, breakthrough behaviour, long-term operation, sorbent regeneration or replacement, sanitation, elemental leaching, sensory acceptability, and spent-sorbent management.

## Figures and Tables

**Figure 1:**
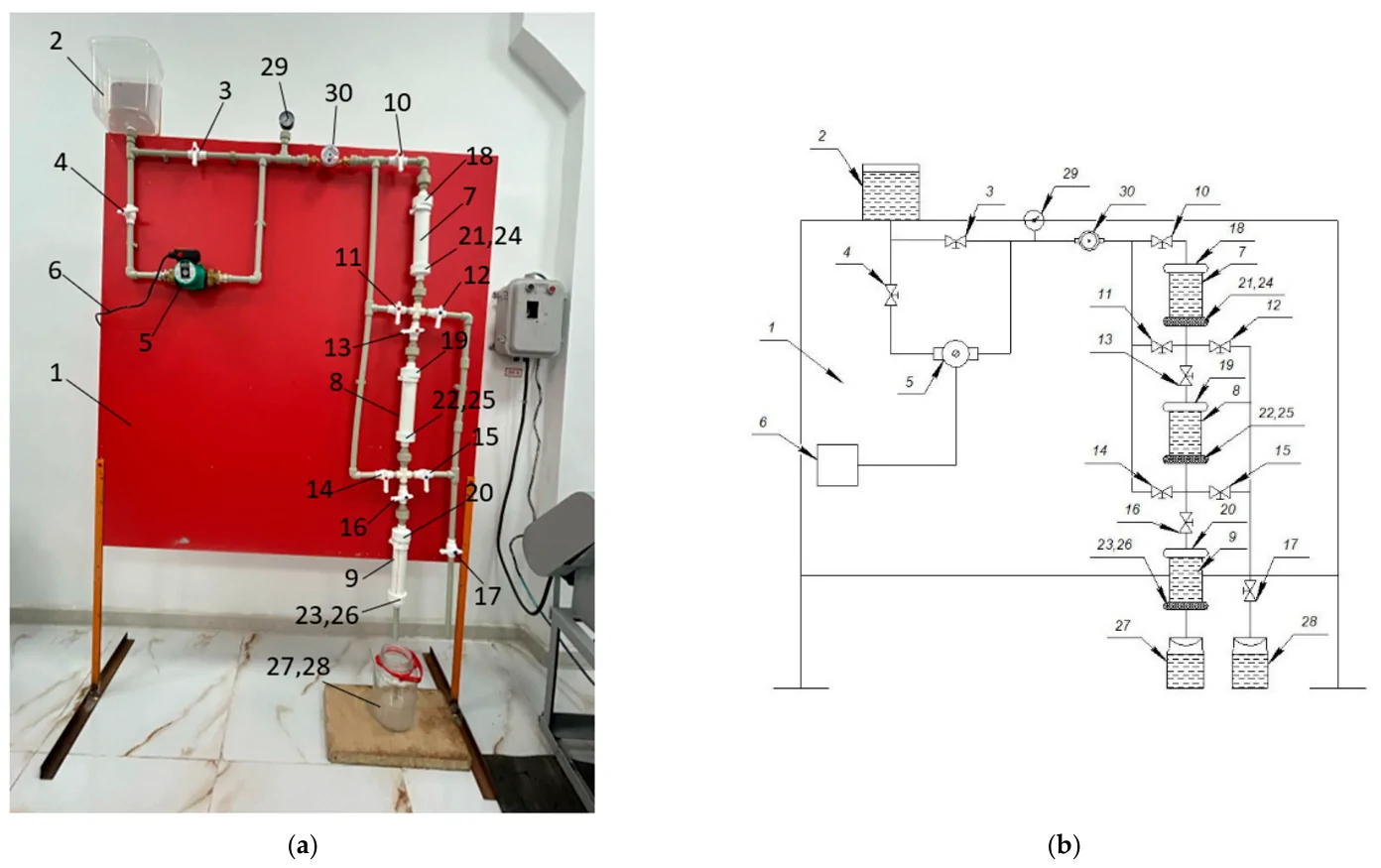
Experimental multistage adsorption–filtration unit for the purification of liquid food products from radionuclides and heavy metals: (**a**) general view of the unit; (**b**) schematic diagram of the unit. 1—Support structure; 2—receiving vessel; 3—gravity-flow mode valve; 4—forced-flow mode valve; 5—centrifugal-impeller pump; 6—connection to the power supply; 7–9—sorption columns; 10–17—control valves; 18–23—rubber gaskets made of heat-/frost-/acid-/alkali-resistant technical rubber; 24–26—meshes to prevent sorbent carryover; 27, 28—collection vessels; 29—manometer; 30—flowmeter.

**Figure 2:**
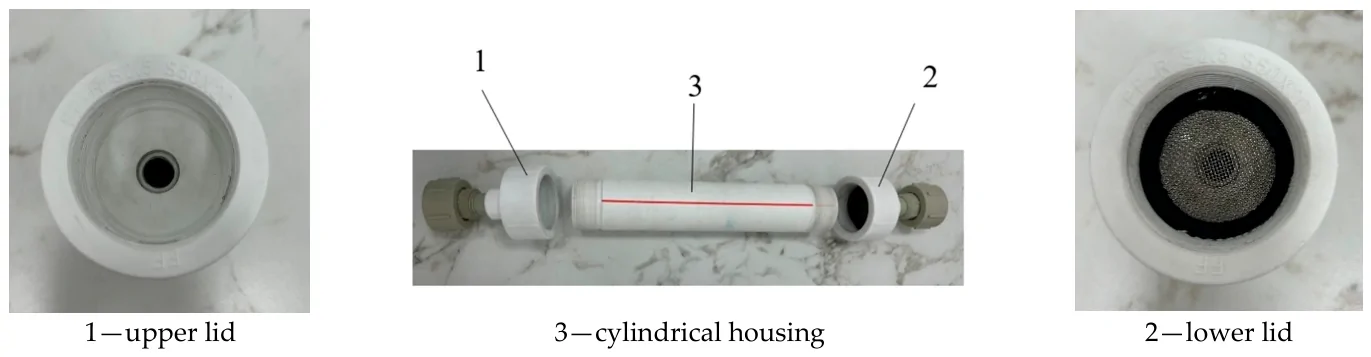
Design of the modular adsorption column with a filtering mesh.

**Figure 3:**
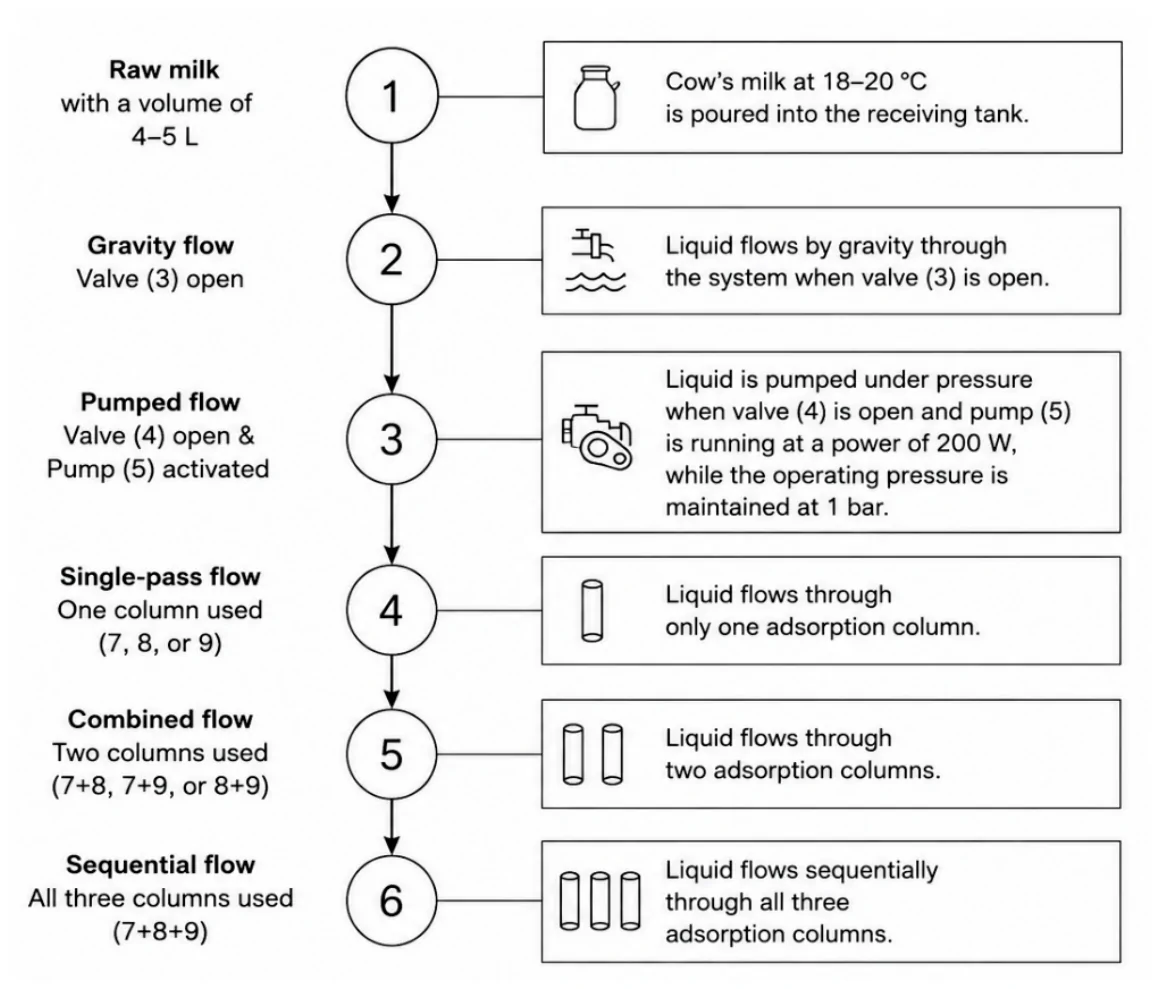
Scheme of liquid flow modes in the purification system.

**Figure 4:**
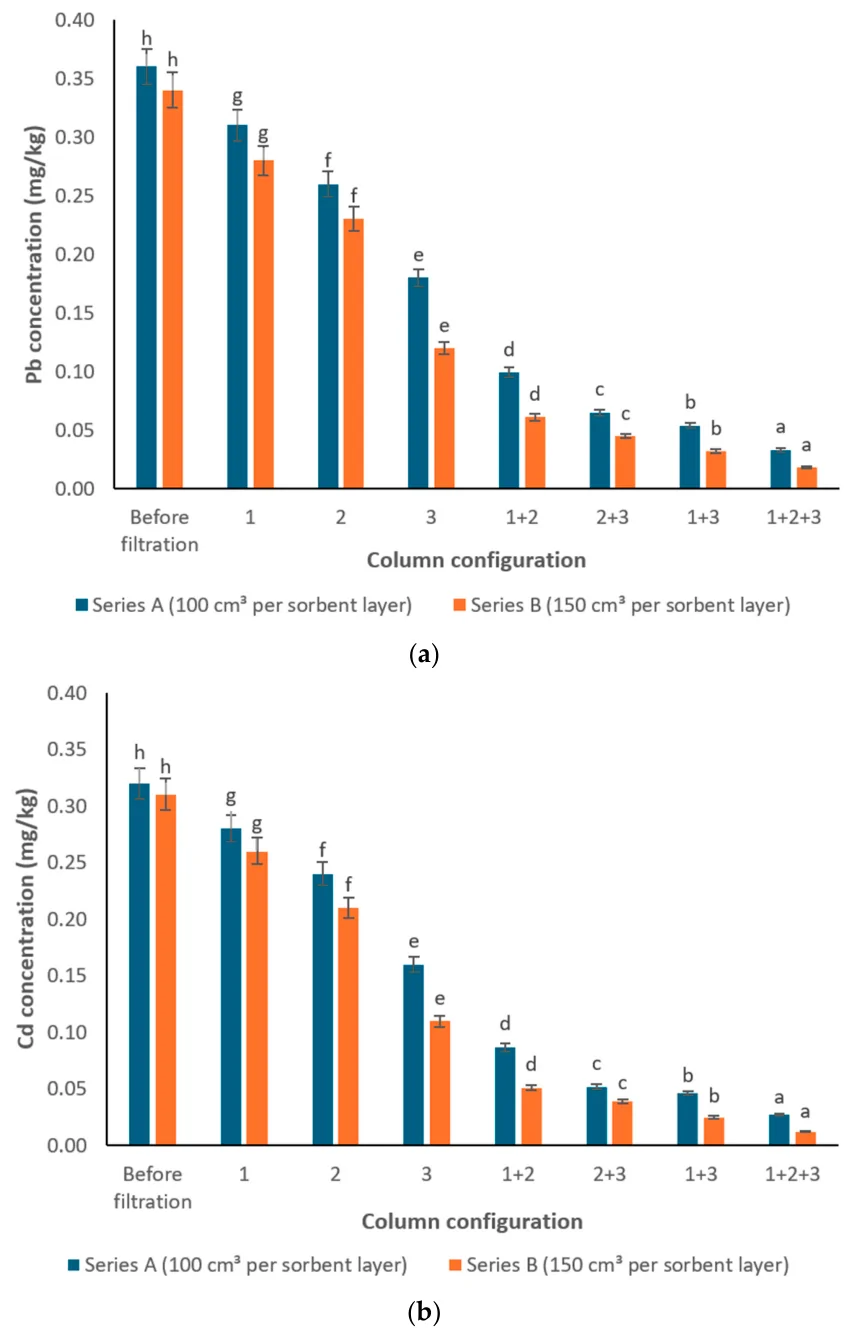
Pb (**a**) and Cd (**b**) concentrations in cow milk before and after sorbent-based filtration using different column configurations in the independently prepared 100 and 150 cm^3^-per-layer series. Different lowercase letters indicate significant differences among column configurations within each loading series (*p* < 0.05); the letters do not indicate comparisons between the two loading series. Because different milk batches were used for the 100 and 150 cm^3^ series, numerical differences between them cannot be attributed exclusively to sorbent loading.

**Figure 5:**
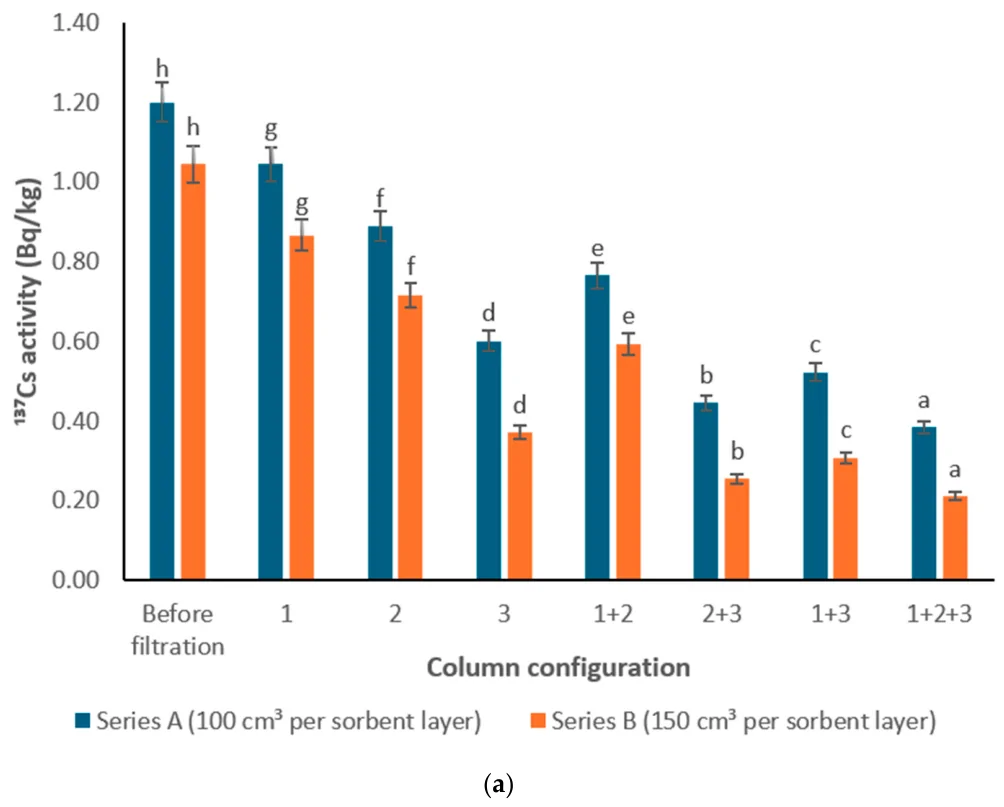

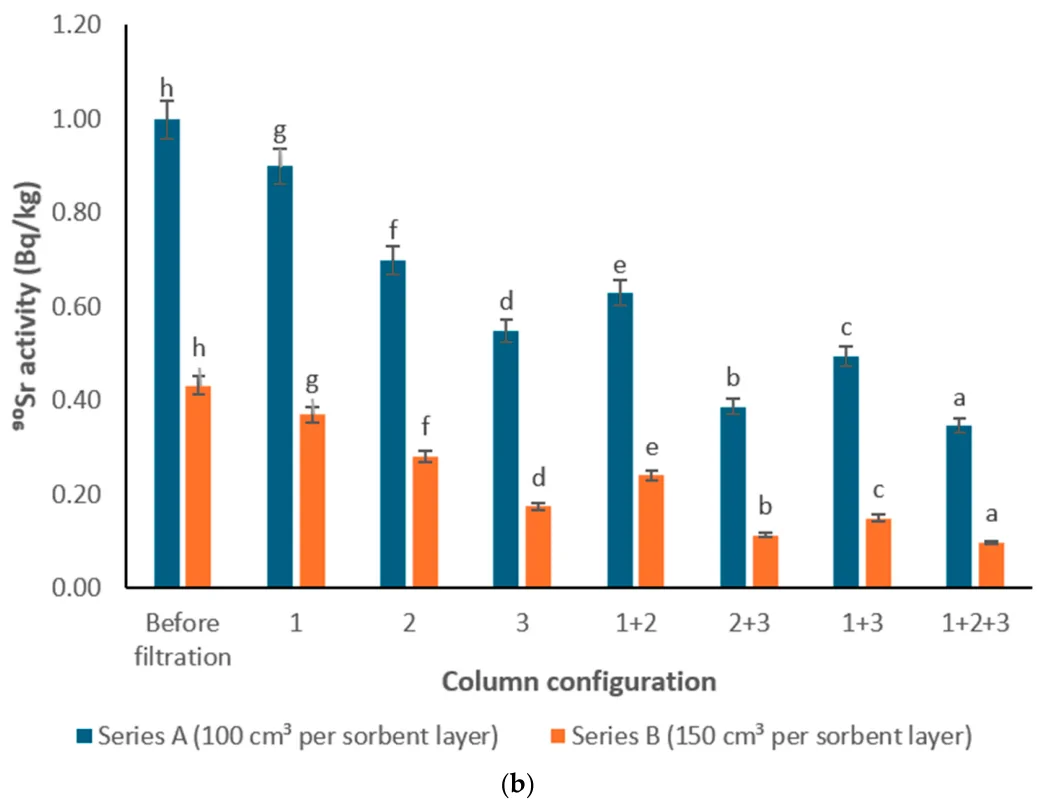
Specific activities of ^137^Cs (**a**) and ^90^Sr (**b**) in cow milk before and after filtration through different sorbent configurations: 1, taurite; 2, natural zeolite; 3, activated carbon 207C; 1 + 2, taurite→natural zeolite; 2 + 3, natural zeolite→activated carbon 207C; 1 + 3, taurite→activated carbon 207C; and 1 + 2 + 3, taurite→natural zeolite→activated carbon 207C. Series A represents Kainar milk treated with 100 cm^3^ per sorbent layer, whereas Series B represents Sarzhal milk treated with 150 cm^3^ per layer. Different lowercase letters indicate significant differences among column configurations within each series (*p* < 0.05); the letters do not indicate comparisons between Series A and Series B.

**Figure 6:**
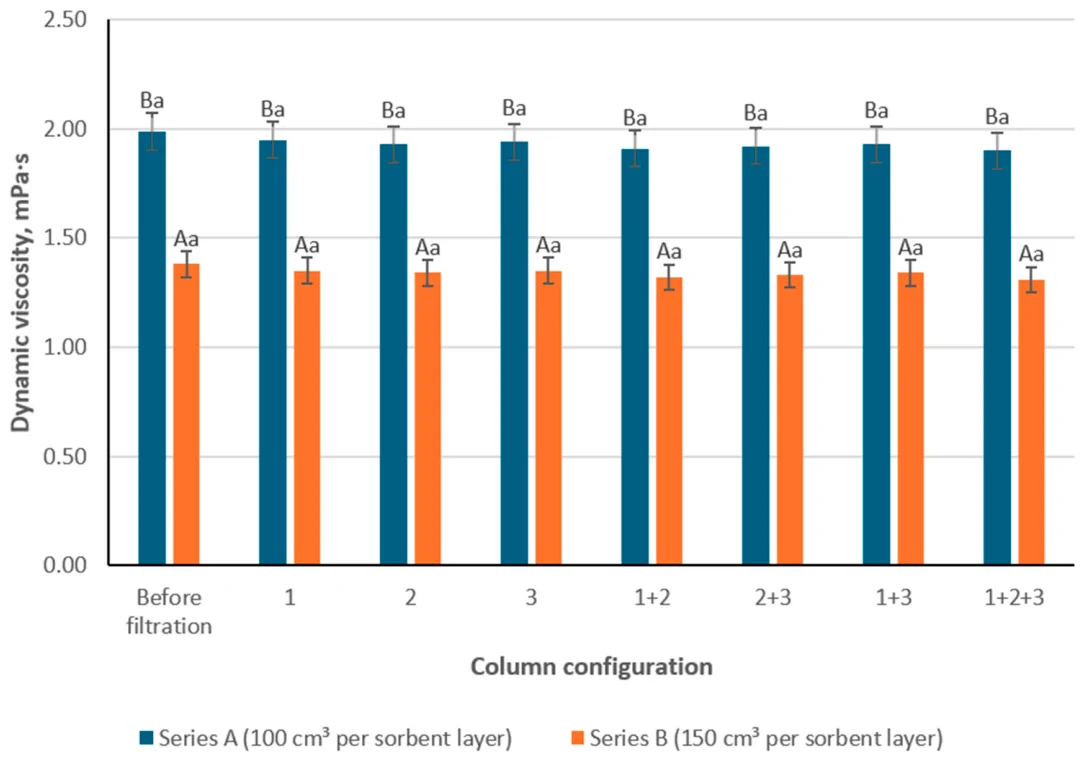
Dynamic viscosity of cow milk before and after adsorption–filtration using different column configurations at sorbent loadings of 100 and 150 cm^3^ per layer. Different uppercase letters indicate significant differences between Series A and Series B within the same column configuration, whereas the same lowercase letter indicates no significant differences among column configurations within the same experimental series (*p* > 0.05).

**Figure 7:**
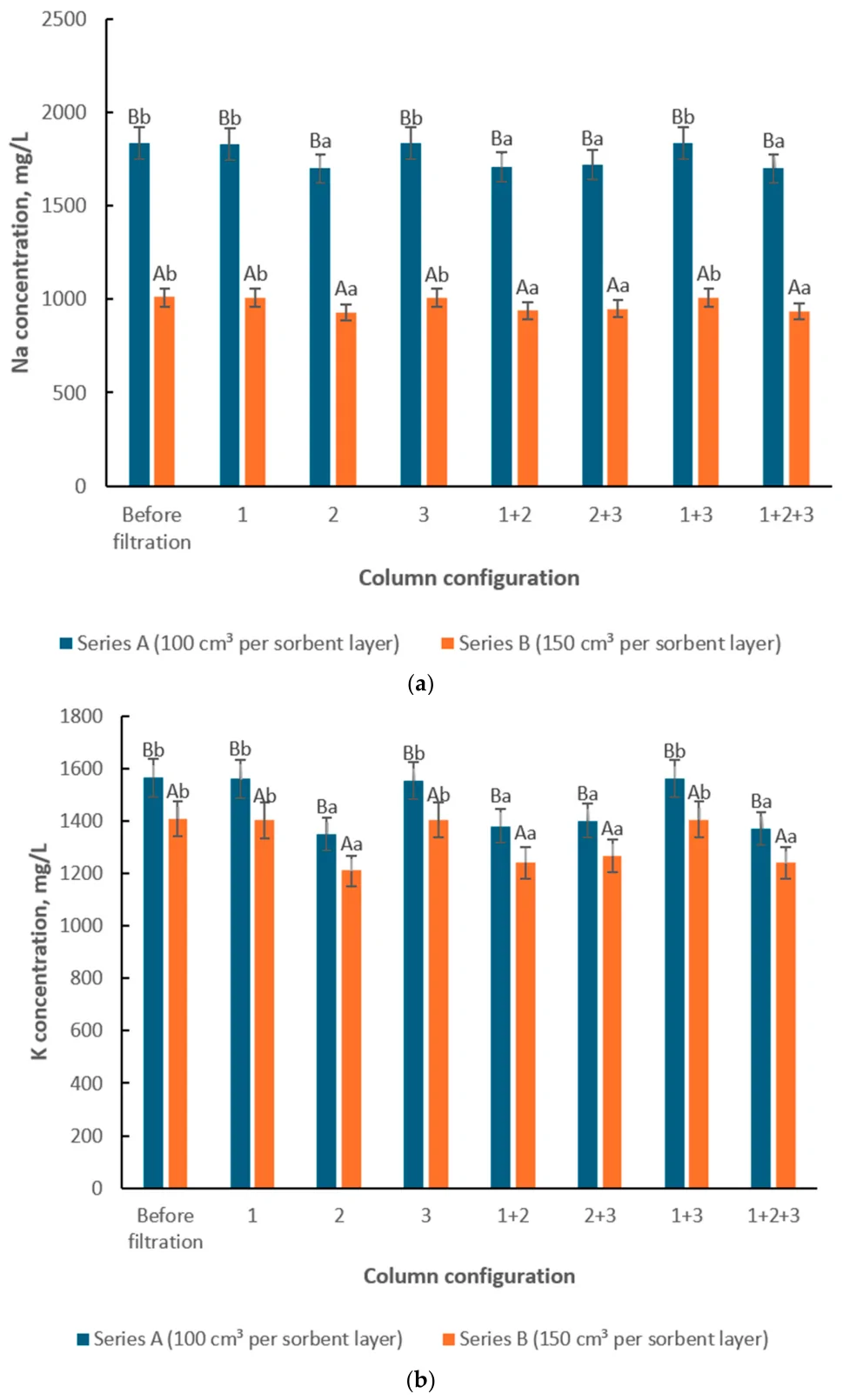

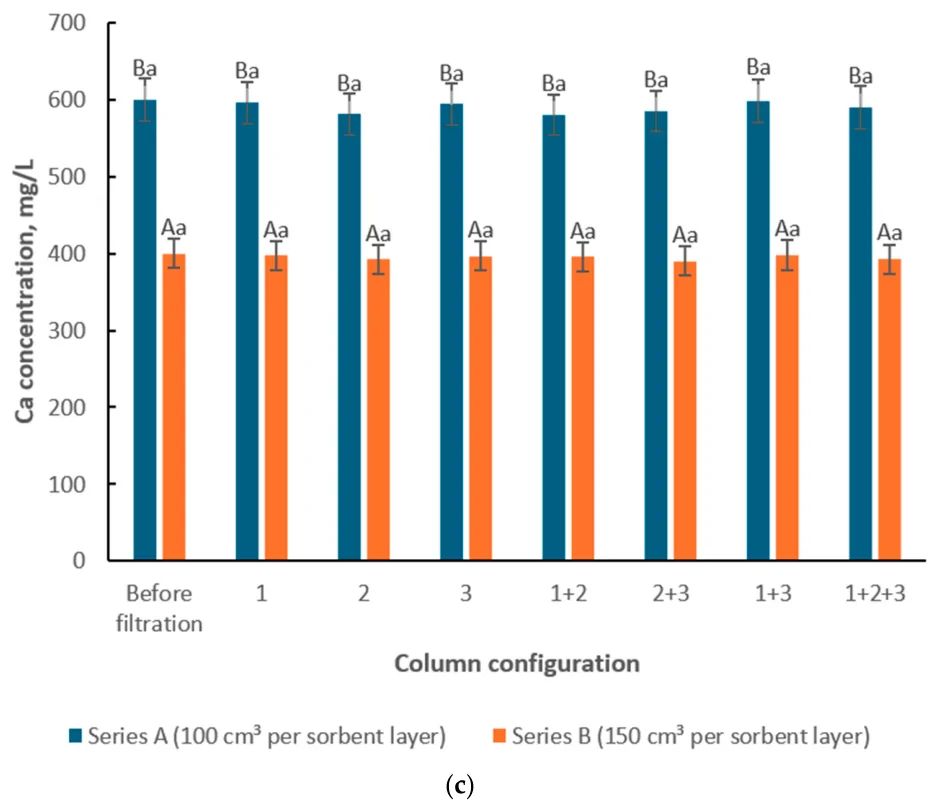
Concentrations of Na (**a**), K (**b**), and Ca (**c**) in cow milk before and after adsorption–filtration using different column configurations at sorbent loadings of 100 and 150 cm^3^ per layer. Different uppercase letters indicate significant differences between Series A and Series B within the same column configuration. In contrast, different lowercase letters indicate significant differences among column configurations within the same experimental series (*p* < 0.05).

**Table 1:** Supplier-reported mineralogical, chemical, and physical characteristics of the sorbent products used in the study.

Sorbent	Origin and Raw Material	Predominant Mineralogical/ Structural Components *	Chemical Composition (wt.%) *	Bulk Density (g/cm^3^)	Moisture (%)	Specific Surface Area (m^2^/g)	External Appearance	Particle Size Used (mm)
Taurite TK	Koksu deposit, Almaty Region, Kazakhstan; taurite-bearing raw material	Calcite, quartz, sericite, and carbonaceous matter	C, 7.0–15.0; SiO_2_, 30.0–55.0; CaO, 9.36; Al_2_O_3_, 9.41; Fe_2_O_3_, 4.83; K_2_O, 2.19; MgO, 1.44; TiO_2_, 0.89; Na_2_O, 0.40	0.60	≤1.0	n.a.	Black carbon–mineral granules	1–2
Natural zeolite	Chankanai deposit, Almaty Region, Kazakhstan	Clinoptilolite (predominant), quartz, and feldspars	SiO_2_, 60–74; Al_2_O_3_, 14–15; CaO, 0.2–1.2; MgO, 0.13–6.40; Fe_2_O_3_, 1.40–5.83; FeO, 0.067–0.199; TiO_2_, 0.07–0.70; K_2_O, 0.66–4.03; Na_2_O, 0.61–5.45; P_2_O_5_, 0.012–0.173	1.17–1.32	n.a.	420	Reddish-brown to gray granules	1–2
Activated carbon 207C	Coconut shells; Ust-Kamenogorsk, East Kazakhstan Region, Kazakhstan	Porous carbonaceous structure	C, 80–90; O, 5–15; H, 0.5–1.5; N, ≤0.5; S, ≤0.1; SiO_2_, 0.5–1.5; Al_2_O_3_, 0.5–1.0; CaO, 0.3–0.7; MgO, 0.2–0.5; Fe_2_O_3_, 0.1–0.5; K_2_O + Na_2_O, 0.2–0.6	0.49–0.53	≤5.0	1050–1150	Black porous granules	1–2

* Chemical compositions are reported on a weight basis according to the supplier’s quality documentation for the sorbent batches used. n.a., not available.

**Table 2:** Physicochemical characteristics of cow milk before and after adsorption–filtration using different column configurations.

Column Configuration	Fat (%)	Lactose (%)	Protein (%)	Total Solids (%)	SNF (%)	Density (kg/m^3^)	pH
Series A: 100 cm^3^ per sorbent layer							
Before filtration	3.31 ± 0.05 ^a^	4.50 ± 0.08 ^a^	3.05 ± 0.06 ^ab^	11.56 ± 0.26 ^ab^	8.25 ± 0.17 ^a^	1028.3 ± 2.0 ^a^	6.62 ± 0.13 ^a^
Taurite	3.28 ± 0.05 ^a^	4.47 ± 0.07 ^a^	3.03 ± 0.05 ^a^	11.50 ± 0.24 ^ab^	8.23 ± 0.16 ^a^	1028.2 ± 2.3 ^a^	6.55 ± 0.13 ^a^
Natural zeolite	3.55 ± 0.05 ^b^	4.35 ± 0.09 ^a^	3.25 ± 0.06 ^b^	11.90 ± 0.28 ^ab^	8.47 ± 0.15 ^a^	1029.3 ± 1.3 ^a^	6.41 ± 0.11 ^a^
Activated carbon 207C	3.27 ± 0.04 ^a^	4.46 ± 0.11 ^a^	3.04 ± 0.06 ^ab^	11.35 ± 0.18 ^a^	8.22 ± 0.17 ^a^	1028.3 ± 1.6 ^a^	6.52 ± 0.09 ^a^
Taurite→natural zeolite	3.61 ± 0.06 ^b^	4.32 ± 0.11 ^a^	3.22 ± 0.04 ^ab^	12.05 ± 0.23 ^b^	8.52 ± 0.14 ^a^	1029.7 ± 2.1 ^a^	6.38 ± 0.07 ^a^
Natural zeolite→activated carbon 207C	3.57 ± 0.04 ^b^	4.33 ± 0.09 ^a^	3.17 ± 0.07 ^ab^	11.95 ± 0.17 ^b^	8.43 ± 0.18 ^a^	1029.3 ± 2.1 ^a^	6.35 ± 0.10 ^a^
Taurite→activated carbon 207C	3.29 ± 0.04 ^a^	4.49 ± 0.11 ^a^	3.02 ± 0.05 ^a^	11.32 ± 0.14 ^a^	8.21 ± 0.12 ^a^	1028.2 ± 2.1 ^a^	6.52 ± 0.12 ^a^
Taurite→natural zeolite→activated carbon 207C	3.63 ± 0.06 ^b^	4.28 ± 0.09 ^a^	3.25 ± 0.06 ^b^	12.15 ± 0.27 ^b^	8.54 ± 0.15 ^a^	1029.6 ± 2.0 ^a^	6.32 ± 0.10 ^a^
Series B: 150 cm^3^ per sorbent layer							
Before filtration	3.28 ± 0.04 ^a^	4.46 ± 0.09 ^a^	3.03 ± 0.05 ^a^	11.46 ± 0.15 ^ab^	8.18 ± 0.15 ^a^	1028.1 ± 1.3 ^a^	6.51 ± 0.11 ^a^
Taurite	3.25 ± 0.06 ^a^	4.43 ± 0.07 ^a^	3.01 ± 0.05 ^a^	11.40 ± 0.16 ^a^	8.13 ± 0.12 ^a^	1028.0 ± 1.5 ^a^	6.39 ± 0.08 ^a^
Natural zeolite	3.54 ± 0.07 ^b^	4.26 ± 0.11 ^a^	3.12 ± 0.04 ^a^	11.82 ± 0.22 ^ab^	8.35 ± 0.15 ^a^	1029.5 ± 1.5 ^a^	6.19 ± 0.15 ^a^
Activated carbon 207C	3.23 ± 0.04 ^a^	4.42 ± 0.10 ^a^	3.00 ± 0.05 ^a^	11.30 ± 0.22 ^a^	8.12 ± 0.10 ^a^	1028.0 ± 1.6 ^a^	6.36 ± 0.08 ^a^
Taurite→natural zeolite	3.60 ± 0.04 ^b^	4.33 ± 0.07 ^a^	3.21 ± 0.06 ^a^	11.96 ± 0.16 ^b^	8.44 ± 0.20 ^a^	1029.5 ± 1.3 ^a^	6.34 ± 0.11 ^a^
Natural zeolite→activated carbon 207C	3.55 ± 0.05 ^b^	4.37 ± 0.09 ^a^	3.19 ± 0.07 ^a^	11.90 ± 0.24 ^b^	8.44 ± 0.12 ^a^	1029.3 ± 1.8 ^a^	6.32 ± 0.15 ^a^
Taurite→activated carbon 207C	3.24 ± 0.04 ^a^	4.44 ± 0.07 ^a^	3.02 ± 0.07 ^a^	11.28 ± 0.23 ^a^	8.16 ± 0.16 ^a^	1028.0 ± 1.7 ^a^	6.41 ± 0.11 ^a^
Taurite→natural zeolite→activated carbon 207C	3.61 ± 0.04 ^b^	4.32 ± 0.04 ^a^	3.22 ± 0.05 ^a^	12.06 ± 0.28 ^b^	8.51 ± 0.17 ^a^	1029.5 ± 2.0 ^a^	6.30 ± 0.10 ^a^

Values are presented as mean ± SD of three independent milk batches (*n* = 3). Within each experimental series and for each physicochemical parameter, different lowercase superscript letters indicate significant differences among column configurations (*p* < 0.05), whereas values sharing the same lowercase letter are not significantly different (*p* > 0.05). No significant differences were observed between Series A (100 cm^3^ per sorbent layer) and Series B (150 cm^3^ per sorbent layer) for the corresponding physicochemical parameters (*p* > 0.05). The freezing point remained unchanged within each experimental series (−0.529 °C for Series A and −0.525 °C for Series B), no added water was detected, and all measurements were performed at a milk temperature of 25 °C.

**Table 3:** Comparative performance of column configurations for heavy metal and radionuclide removal and preservation of milk mineral composition at a sorbent loading of 150 cm^3^ per layer.

Column Configuration	Mean Pb/Cd Removal (%)	Final Pb/Cd (mg/kg)	Pb/Cd Regulatory Compliance ^1^	^137^Cs Removal (%)	^90^Sr Removal (%)	Mean Mineral Retention ^2^ (%)	Filtration Time(s)
1: Taurite	16.89	0.280/0.260	Neither	17.05	14.23	99.53	10
2: Natural zeolite	32.30	0.230/0.210	Neither	31.51	35.10	91.97	11
3: Activated carbon 207C	64.61	0.120/0.110	Neither	64.56	59.90	99.45	12
1 + 2: Taurite→natural zeolite	82.81	0.061/0.051	Pb only	43.19	44.34	93.34	16
2 + 3: Natural zeolite→activated carbon 207C	87.09	0.045/0.039	Pb only	75.73	73.97	93.81	13
1 + 3: Taurite→activated carbon 207C	91.26	0.032/0.025	Pb and Cd	70.60	65.60	99.71	15
1 + 2 + 3: Taurite→natural zeolite→activated carbon 207C	95.42	0.018/0.012	Pb and Cd	79.87	77.68	92.93	22

^1^ Regulatory compliance for Pb and Cd was evaluated against the maximum permissible concentrations for raw milk specified in TR CU 021/2011: Pb, 0.10 mg/kg; Cd, 0.03 mg/kg. ^2^ Mean mineral retention was calculated as the arithmetic mean of Na, K, and Ca concentration-retention percentages relative to untreated milk.

## Data Availability

The original contributions presented in this study are included in the article. Further inquiries can be directed to the corresponding author.
